# Supplementary material for: Investigating colonization patterns of the infant gut microbiome during the introduction of solid food and weaning from breastmilk: A cohort study protocol
Source: PLoS One. 2021 Apr 2;16(4):e0248924. doi: 10.1371/journal.pone.0248924 (PMC8018627; doi:10.1371/journal.pone.0248924)
Supplement: S1 Appendix — (DOCX) [file pone.0248924.s001.docx]

**S1 Appendix. Study Package**

The study package will consist of:

- - One study diary
  - 17 sample bags (Ziploc^®^, sealable bag 6.5in x 5.86in; Costco Canada, 105134) with labels containing participant identification number, the study day (Day 1-17), a space for the date of sample collection, and a checkbox for if the sample was collected before/the day of/or after introduction of solids/weaning from breastmilk
  - Two anaerobic sachets (Thermo Scientific™, Oxoid™, Anaerogen™, 2.5L Sachet; Fisher Scientific, OXAN0025A)
  - One small insulated bag (Cooking Concepts™, Insulated Bag 8in (L) x 4in (H) x 5in (W); Dollar Tree, 3927727314)
  - One small ice/cooler pack
  - One large insulated bag (Thermal bag 19in x 19in; Dollar Tree^®^, 197260)
  - One large ice/cooler pack
  - One Eppendorf tube for collection of diaper cream (1.7mL microcentrifuge tube; VWR^®^, 87003-294)
  - One pack of diaper liners (100 Bio-Soft Liners 7.5in x 12in; Bummis™)
