# Supplementary material for: Investigating colonization patterns of the infant gut microbiome during the introduction of solid food and weaning from breastmilk: A cohort study protocol
Source: PLoS One. 2021 Apr 2;16(4):e0248924. doi: 10.1371/journal.pone.0248924 (PMC8018627; doi:10.1371/journal.pone.0248924)
Supplement: S2 Appendix — (PDF) [file pone.0248924.s002.pdf]

Introduction of Solid Food Diary

1. Participant ID	□□-□□□
2. Infant birth date	day □□ month □□ 20□□
3. Height of infant	□□□.□ cm<br>OR<br>□□.□ inches
4. Weight of infant	□□.□ kg<br>OR<br>□□ pounds and □□ ounces

Instructions

1. **During the 17 day study period, note as detailed as possible:**
    - What your child eats or drinks;
    - How much your child ate or drank;
    - During which time of the day.

2. **Note as precisely as possible what your child eats or drinks.**
    - For example, note the brand of the food product or how fresh food products were processed.

3. **Note everything, including:**
    - Small bites or nips, biscuits;
    - If bread is smeared;
    - The use of vitamin supplements
    - The processing of the food (e.g. cooked, boiled, mashed, peeled, blended)

4. **Note the amounts in portions**
    - For example: ½ mashed fresh banana, 200ml formula, 200 ml breast milk, 10 minutes at the breast, half a biscuit, 2 tablespoons of peeled, boiled and mashed potato

5. **Note how well your child tolerated the food** (spitting up, cramps/colic)

6. **Note the number of bowel movement for each of the days**

7. **Note the stool consistency of the collected fecal samples**
    - Refer to Bristol Stool Chart located at back of booklet

Example

Date	day 2 1 month 0 8 20 1 6
Infant fecal sample collected	☒ yes ☐ no
Stool consistency of collected infant fecal sample (see Bristol Stool Chart)	☐ Type 1 ☐ Type 2 ☒ Type 3<br>☐ Type 4 ☐ Type 5 ☐ Type 6 ☐ Type 7
Time of collected stool sample	0 2 : 0 5 ☐ 24 hour clock ☐ AM ☒ PM
Did this sample come into contact with diaper cream?	☒ yes, brand: homemade: coconut oil and beeswax ☐ no
This sample was collected from a (select one):	☐ disposable diaper ☒ cloth diaper ☐ potty ☐ toilet ☐ other
Number of bowel movements during this day	0 2 bowel movements
Other comments (e.g. infections, health complaints)	She has a cold.
If you are currently breastfeeding, please list any prescription medications that you took today.	Synthroid (levothyroxine)

How much time did your baby spend in sleep last night between 7pm and 7am?	1 0 hours and 4 5 minutes
How many times did your baby wake up last night (7pm to 7am)?	0 3 times
How much time did your baby spend in wakefulness between 10pm and 6am?	0 1 hours and 1 5 minutes

Please indicate if your baby had any interaction today with:	☒ Siblings ☐ Other children ☒ Animals that stay inside the house<br>☐ Animals that go outside and inside the house ☐ Animals that stay outside the house

Example

	Quantity	Food or Drink	Processing	Brand	Tolerated?
**Feeding 1**<br>Time:<br>3:00 am	____	Breastfeeding ~ 10 minutes both sides	____	____	Yes – fell asleep at breast
**Feeding 2**<br>Time:<br>6:30 am	____	Breastfeeding ~ 10 minutes both sides	____	____	yes
**Feeding 3**<br>Time:<br>9:00 am	1 tbsp	Rice cereal	Mixed with expressed breast milk	President's Choice	Seemed to like it better today than last time we tried!
**Feeding 4**<br>Time:<br>12:00 pm	____	Breastfeeding ~ 5 minutes both sides	____	____	Yes – fell asleep at breast – put down for nap
**Feeding 5**<br>Time:<br>3:00 pm	1	Rice Rusk	____	Hot-Kid Baby Mum Mum	She loves these!
**Feeding 6**<br>Time:<br>5:30 pm	3 tbsp	Butternut squash puree	Warmed	Homemade	Her favourite food so far!!
	2 tbsp	Green bean puree	Warmed	President's Choice Organic	First time – she didn't like them – spit them out!
**Feeding 7**<br>Time:<br>7:00 pm	____	Breastfeeding ~ 10 minutes both sides	____	____	Yes – fell asleep at breast
**Feeding 8**<br>Time:<br>10:00 pm	____	Breastfeeding ~ 2 minutes both sides	____	____	Yes – just needed to be settled.

Example

	Quantity	Name	Number of doses	Brand	Route (oral, IV, topical etc)
**Medication or vitamin 1**	2.5 ml	Acetaminophen	2	Children's Tylenol	oral
**Medication or vitamin 2**	1 drop	Vitamin D	1	Ddrops	oral
**Medication or vitamin 3**					
**Medication or vitamin 4**					
**Medication or vitamin 5**					

Day 1

Date	day ☐☐ month ☐☐ 20☐☐
Infant fecal sample collected	☐ yes ☐ no
Stool consistency of collected infant fecal sample (see Bristol Stool Chart)	☐ Type 1 ☐ Type 2 ☐ Type 3<br>☐ Type 4 ☐ Type 5 ☐Type 6 ☐ Type 7
Time of collected stool sample	☐☐:☐☐ ☐ 24 hour clock ☐ AM ☐ PM
Did this sample come into contact with diaper cream?	☐ yes, brand:___________________________ ☐ no
This sample was collected from a (select one):	☐ disposable diaper ☐ cloth diaper ☐ potty ☐ toilet ☐ other
Number of bowel movements during this day	☐☐ bowel movements
Other comments (e.g. infections, health complaints)	
If you are currently breastfeeding, please list any prescription medications that you took today.	
How much time did your baby spend in sleep last night between 7pm and 7am?	☐☐ hours and ☐☐ minutes
How many times did your baby wake up last night (7pm to 7am)?	☐☐ times
How much time did your baby spend in wakefulness between 10pm and 6am?	☐☐ hours and ☐☐ minutes
Please indicate if your baby had any interaction today with:	☐ Siblings ☐ Other children ☐ Animals that stay inside the house<br>☐ Animals that go outside and inside the house ☐Animals that stay outside the house

Day 1

	Quantity	Food or Drink	Processing	Brand	Tolerated?
**Feeding 1**<br><br>Time:					
**Feeding 2**<br><br>Time:					
**Feeding 3**<br><br>Time:					
**Feeding 4**<br><br>Time:					
**Feeding 5**<br><br>Time:					
**Feeding 6**<br><br>Time:					

Day 1

	Quantity	Food or Drink	Processing	Brand	Tolerated?
**Feeding 7**<br><br>Time:					
**Feeding 8**<br><br>Time:					
**Feeding 9**<br><br>Time:					

	Quantity	Name	Number of doses	Brand	Route (oral, IV, topical etc)
**Medication or vitamin 1**					
**Medication or vitamin 2**					
**Medication or vitamin 3**					
**Medication or vitamin 4**					
**Medication or vitamin 5**					

Day 2

Date	day ☐☐ month ☐☐ 20☐☐
Infant fecal sample collected	☐ yes ☐ no
Stool consistency of collected infant fecal sample (see Bristol Stool Chart)	☐ Type 1 ☐ Type 2 ☐ Type 3<br>☐ Type 4 ☐ Type 5 ☐Type 6 ☐ Type 7
Time of collected stool sample	☐☐:☐☐ ☐ 24 hour clock ☐ AM ☐ PM
Did this sample come into contact with diaper cream?	☐ yes, brand:___________________________ ☐ no
This sample was collected from a (select one):	☐ disposable diaper ☐ cloth diaper ☐ potty ☐ toilet ☐ other
Number of bowel movements during this day	☐☐ bowel movements
Other comments (e.g. infections, health complaints)	
If you are currently breastfeeding, please list any prescription medications that you took today.	
How much time did your baby spend in sleep last night between 7pm and 7am?	☐☐ hours and ☐☐ minutes
How many times did your baby wake up last night (7pm to 7am)?	☐☐ times
How much time did your baby spend in wakefulness between 10pm and 6am?	☐☐ hours and ☐☐ minutes
Please indicate if your baby had any interaction today with:	☐ Siblings ☐ Other children ☐ Animals that stay inside the house<br>☐ Animals that go outside and inside the house ☐Animals that stay outside the house

Day 2

	Quantity	Food or Drink	Processing	Brand	Tolerated?
**Feeding 1**<br><br>Time:					
**Feeding 2**<br><br>Time:					
**Feeding 3**<br><br>Time:					
**Feeding 4**<br><br>Time:					
**Feeding 5**<br><br>Time:					
**Feeding 6**<br><br>Time:					

Day 2

	Quantity	Food or Drink	Processing	Brand	Tolerated?
**Feeding 7**<br><br>Time:					
**Feeding 8**<br><br>Time:					
**Feeding 9**<br><br>Time:					

	Quantity	Name	Number of doses	Brand	Route (oral, IV, topical etc)
**Medication or vitamin 1**					
**Medication or vitamin 2**					
**Medication or vitamin 3**					
**Medication or vitamin 4**					
**Medication or vitamin 5**					

Day 3

Date	day ☐☐ month ☐☐ 20☐☐
Infant fecal sample collected	☐ yes ☐ no
Stool consistency of collected infant fecal sample (see Bristol Stool Chart)	☐ Type 1 ☐ Type 2 ☐ Type 3<br>☐ Type 4 ☐ Type 5 ☐Type 6 ☐ Type 7
Time of collected stool sample	☐☐:☐☐ ☐ 24 hour clock ☐ AM ☐ PM
Did this sample come into contact with diaper cream?	☐ yes, brand:___________________________ ☐ no
This sample was collected from a (select one):	☐ disposable diaper ☐ cloth diaper ☐ potty ☐ toilet ☐ other
Number of bowel movements during this day	☐☐ bowel movements
Other comments (e.g. infections, health complaints)	
If you are currently breastfeeding, please list any prescription medications that you took today.	

How much time did your baby spend in sleep last night between 7pm and 7am?	☐☐ hours and ☐☐ minutes
How many times did your baby wake up last night (7pm to 7am)?	☐☐ times
How much time did your baby spend in wakefulness between 10pm and 6am?	☐☐ hours and ☐☐ minutes
Please indicate if your baby had any interaction today with:	☐ Siblings ☐ Other children ☐ Animals that stay inside the house<br>☐ Animals that go outside and inside the house ☐Animals that stay outside the house

Day 3

	Quantity	Food or Drink	Processing	Brand	Tolerated?
**Feeding 1**<br><br>Time:					
**Feeding 2**<br><br>Time:					
**Feeding 3**<br><br>Time:					
**Feeding 4**<br><br>Time:					
**Feeding 5**<br><br>Time:					
**Feeding 6**<br><br>Time:					

Day 3

	Quantity	Food or Drink	Processing	Brand	Tolerated?
**Feeding 7**<br><br>Time:					
**Feeding 8**<br><br>Time:					
**Feeding 9**<br><br>Time:					

	Quantity	Name	Number of doses	Brand	Route (oral, IV, topical etc)
**Medication or vitamin 1**					
**Medication or vitamin 2**					
**Medication or vitamin 3**					
**Medication or vitamin 4**					
**Medication or vitamin 5**					

Day 4

Date	day ☐☐ month ☐☐ 20☐☐
Infant fecal sample collected	☐ yes ☐ no
Stool consistency of collected infant fecal sample (see Bristol Stool Chart)	☐ Type 1 ☐ Type 2 ☐ Type 3<br>☐ Type 4 ☐ Type 5 ☐Type 6 ☐ Type 7
Time of collected stool sample	☐☐:☐☐ ☐ 24 hour clock ☐ AM ☐ PM
Did this sample come into contact with diaper cream?	☐ yes, brand:___________________________ ☐ no
This sample was collected from a (select one):	☐ disposable diaper ☐ cloth diaper ☐ potty ☐ toilet ☐ other
Number of bowel movements during this day	☐☐ bowel movements
Other comments (e.g. infections, health complaints)	
If you are currently breastfeeding, please list any prescription medications that you took today.	
How much time did your baby spend in sleep last night between 7pm and 7am?	☐☐ hours and ☐☐ minutes
How many times did your baby wake up last night (7pm to 7am)?	☐☐ times
How much time did your baby spend in wakefulness between 10pm and 6am?	☐☐ hours and ☐☐ minutes
Please indicate if your baby had any interaction today with:	☐ Siblings ☐ Other children ☐ Animals that stay inside the house<br>☐ Animals that go outside and inside the house ☐Animals that stay outside the house

Day 4

	Quantity	Food or Drink	Processing	Brand	Tolerated?
**Feeding 1**					
Time:					
**Feeding 2**					
Time:					
**Feeding 3**					
Time:					
**Feeding 4**					
Time:					
**Feeding 5**					
Time:					
**Feeding 6**					
Time:					

Day 4

	Quantity	Food or Drink	Processing	Brand	Tolerated?
**Feeding 7**<br><br>Time:					
**Feeding 8**<br><br>Time:					
**Feeding 9**<br><br>Time:					

	Quantity	Name	Number of doses	Brand	Route (oral, IV, topical etc)
**Medication or vitamin 1**					
**Medication or vitamin 2**					
**Medication or vitamin 3**					
**Medication or vitamin 4**					
**Medication or vitamin 5**					

Day 5

Date	day ☐☐ month ☐☐ 20☐☐
Infant fecal sample collected	☐ yes ☐ no
Stool consistency of collected infant fecal sample (see Bristol Stool Chart)	☐ Type 1 ☐ Type 2 ☐ Type 3<br>☐ Type 4 ☐ Type 5 ☐Type 6 ☐ Type 7
Time of collected stool sample	☐☐:☐☐ ☐ 24 hour clock ☐ AM ☐ PM
Did this sample come into contact with diaper cream?	☐ yes, brand:___________________________ ☐ no
This sample was collected from a (select one):	☐ disposable diaper ☐ cloth diaper ☐ potty ☐ toilet ☐ other
Number of bowel movements during this day	☐☐ bowel movements
Other comments (e.g. infections, health complaints)	
If you are currently breastfeeding, please list any prescription medications that you took today.	
How much time did your baby spend in sleep last night between 7pm and 7am?	☐☐ hours and ☐☐ minutes
How many times did your baby wake up last night (7pm to 7am)?	☐☐ times
How much time did your baby spend in wakefulness between 10pm and 6am?	☐☐ hours and ☐☐ minutes
Please indicate if your baby had any interaction today with:	☐ Siblings ☐ Other children ☐ Animals that stay inside the house<br>☐ Animals that go outside and inside the house ☐Animals that stay outside the house

Day 5

	Quantity	Food or Drink	Processing	Brand	Tolerated?
**Feeding 1**<br><br>Time:					
**Feeding 2**<br><br>Time:					
**Feeding 3**<br><br>Time:					
**Feeding 4**<br><br>Time:					
**Feeding 5**<br><br>Time:					
**Feeding 6**<br><br>Time:					

Day 5

	Quantity	Food or Drink	Processing	Brand	Tolerated?
**Feeding 7**<br><br>Time:					
**Feeding 8**<br><br>Time:					
**Feeding 9**<br><br>Time:					

	Quantity	Name	Number of doses	Brand	Route (oral, IV, topical etc)
**Medication or vitamin 1**					
**Medication or vitamin 2**					
**Medication or vitamin 3**					
**Medication or vitamin 4**					
**Medication or vitamin 5**					

Day 6

Date	day ☐☐ month ☐☐ 20☐☐
Infant fecal sample collected	☐ yes ☐ no
Stool consistency of collected infant fecal sample (see Bristol Stool Chart)	☐ Type 1 ☐ Type 2 ☐ Type 3<br>☐ Type 4 ☐ Type 5 ☐Type 6 ☐ Type 7
Time of collected stool sample	☐☐:☐☐ ☐ 24 hour clock ☐ AM ☐ PM
Did this sample come into contact with diaper cream?	☐ yes, brand:___________________________ ☐ no
This sample was collected from a (select one):	☐ disposable diaper ☐ cloth diaper ☐ potty ☐ toilet ☐ other
Number of bowel movements during this day	☐☐ bowel movements
Other comments (e.g. infections, health complaints)	
If you are currently breastfeeding, please list any prescription medications that you took today.	

How much time did your baby spend in sleep last night between 7pm and 7am?	☐☐ hours and ☐☐ minutes
How many times did your baby wake up last night (7pm to 7am)?	☐☐ times
How much time did your baby spend in wakefulness between 10pm and 6am?	☐☐ hours and ☐☐ minutes
Please indicate if your baby had any interaction today with:	☐ Siblings ☐ Other children ☐ Animals that stay inside the house<br>☐ Animals that go outside and inside the house ☐Animals that stay outside the house

Day 6

	Quantity	Food or Drink	Processing	Brand	Tolerated?
**Feeding 1**					
Time:					
**Feeding 2**					
Time:					
**Feeding 3**					
Time:					
**Feeding 4**					
Time:					
**Feeding 5**					
Time:					
**Feeding 6**					
Time:					

Day 6

	Quantity	Food or Drink	Processing	Brand	Tolerated?
**Feeding 7**<br><br>Time:					
**Feeding 8**<br><br>Time:					
**Feeding 9**<br><br>Time:					

	Quantity	Name	Number of doses	Brand	Route (oral, IV, topical etc)
**Medication or vitamin 1**					
**Medication or vitamin 2**					
**Medication or vitamin 3**					
**Medication or vitamin 4**					
**Medication or vitamin 5**					

Day 7

Date	day ☐☐ month ☐☐ 20☐☐
Infant fecal sample collected	☐ yes ☐ no
Stool consistency of collected infant fecal sample (see Bristol Stool Chart)	☐ Type 1 ☐ Type 2 ☐ Type 3<br>☐ Type 4 ☐ Type 5 ☐Type 6 ☐ Type 7
Time of collected stool sample	☐☐:☐☐ ☐ 24 hour clock ☐ AM ☐ PM
Did this sample come into contact with diaper cream?	☐ yes, brand:_________________________ ☐ no
This sample was collected from a (select one):	☐ disposable diaper ☐ cloth diaper ☐ potty ☐ toilet ☐ other
Number of bowel movements during this day	☐☐ bowel movements
Other comments (e.g. infections, health complaints)	
If you are currently breastfeeding, please list any prescription medications that you took today.	

How much time did your baby spend in sleep last night between 7pm and 7am?	☐☐ hours and ☐☐ minutes
How many times did your baby wake up last night (7pm to 7am)?	☐☐ times
How much time did your baby spend in wakefulness between 10pm and 6am?	☐☐ hours and ☐☐ minutes
Please indicate if your baby had any interaction today with:	☐ Siblings ☐ Other children ☐ Animals that stay inside the house<br>☐ Animals that go outside and inside the house ☐Animals that stay outside the house

Day 7

	Quantity	Food or Drink	Processing	Brand	Tolerated?
**Feeding 1**<br>Time:					
**Feeding 2**<br>Time:					
**Feeding 3**<br>Time:					
**Feeding 4**<br>Time:					
**Feeding 5**<br>Time:					
**Feeding 6**<br>Time:					

Day 7

	Quantity	Food or Drink	Processing	Brand	Tolerated?
**Feeding 7**<br><br>Time:					
**Feeding 8**<br><br>Time:					
**Feeding 9**<br><br>Time:					

	Quantity	Name	Number of doses	Brand	Route (oral, IV, topical etc)
**Medication or vitamin 1**					
**Medication or vitamin 2**					
**Medication or vitamin 3**					
**Medication or vitamin 4**					
**Medication or vitamin 5**					

Day 8

Date	day ☐☐ month ☐☐ 20☐☐
Infant fecal sample collected	☐ yes ☐ no
Stool consistency of collected infant fecal sample (see Bristol Stool Chart)	☐ Type 1 ☐ Type 2 ☐ Type 3<br>☐ Type 4 ☐ Type 5 ☐Type 6 ☐ Type 7
Time of collected stool sample	☐☐:☐☐ ☐ 24 hour clock ☐ AM ☐ PM
Did this sample come into contact with diaper cream?	☐ yes, brand:___________________________ ☐ no
This sample was collected from a (select one):	☐ disposable diaper ☐ cloth diaper ☐ potty ☐ toilet ☐ other
Number of bowel movements during this day	☐☐ bowel movements
Other comments (e.g. infections, health complaints)	
If you are currently breastfeeding, please list any prescription medications that you took today.	

How much time did your baby spend in sleep last night between 7pm and 7am?	☐☐ hours and ☐☐ minutes
How many times did your baby wake up last night (7pm to 7am)?	☐☐ times
How much time did your baby spend in wakefulness between 10pm and 6am?	☐☐ hours and ☐☐ minutes

Please indicate if your baby had any interaction today with:	☐ Siblings ☐ Other children ☐ Animals that stay inside the house<br>☐ Animals that go outside and inside the house ☐Animals that stay outside the house

Day 8

	Quantity	Food or Drink	Processing	Brand	Tolerated?
**Feeding 1**<br><br>Time:					
**Feeding 2**<br><br>Time:					
**Feeding 3**<br><br>Time:					
**Feeding 4**<br><br>Time:					
**Feeding 5**<br><br>Time:					
**Feeding 6**<br><br>Time:					

Day 8

	Quantity	Food or Drink	Processing	Brand	Tolerated?
**Feeding 7**					
Time:					
**Feeding 8**					
Time:					
**Feeding 9**					
Time:					

	Quantity	Name	Number of doses	Brand	Route (oral, IV, topical etc)
**Medication or vitamin 1**					
**Medication or vitamin 2**					
**Medication or vitamin 3**					
**Medication or vitamin 4**					
**Medication or vitamin 5**					

Day 9

Date	day ☐☐ month ☐☐ 20☐☐
Infant fecal sample collected	☐ yes ☐ no
Stool consistency of collected infant fecal sample (see Bristol Stool Chart)	☐ Type 1 ☐ Type 2 ☐ Type 3<br>☐ Type 4 ☐ Type 5 ☐Type 6 ☐ Type 7
Time of collected stool sample	☐☐:☐☐ ☐ 24 hour clock ☐ AM ☐ PM
Did this sample come into contact with diaper cream?	☐ yes, brand:___________________________ ☐ no
This sample was collected from a (select one):	☐ disposable diaper ☐ cloth diaper ☐ potty ☐ toilet ☐ other
Number of bowel movements during this day	☐☐ bowel movements
Other comments (e.g. infections, health complaints)	
If you are currently breastfeeding, please list any prescription medications that you took today.	

How much time did your baby spend in sleep last night between 7pm and 7am?	☐☐ hours and ☐☐ minutes
How many times did your baby wake up last night (7pm to 7am)?	☐☐ times
How much time did your baby spend in wakefulness between 10pm and 6am?	☐☐ hours and ☐☐ minutes

Please indicate if your baby had any interaction today with:	☐ Siblings ☐ Other children ☐ Animals that stay inside the house<br>☐ Animals that go outside and inside the house ☐Animals that stay outside the house

Day 9

	Quantity	Food or Drink	Processing	Brand	Tolerated?
**Feeding 1**<br>Time:					
**Feeding 2**<br>Time:					
**Feeding 3**<br>Time:					
**Feeding 4**<br>Time:					
**Feeding 5**<br>Time:					
**Feeding 6**<br>Time:					

Day 9

	Quantity	Food or Drink	Processing	Brand	Tolerated?
**Feeding 7**<br><br>Time:					
**Feeding 8**<br><br>Time:					
**Feeding 9**<br><br>Time:					

	Quantity	Name	Number of doses	Brand	Route (oral, IV, topical etc)
**Medication or vitamin 1**					
**Medication or vitamin 2**					
**Medication or vitamin 3**					
**Medication or vitamin 4**					
**Medication or vitamin 5**					

Day 10

Date	day ☐☐ month ☐☐ 20☐☐
Infant fecal sample collected	☐ yes ☐ no
Stool consistency of collected infant fecal sample (see Bristol Stool Chart)	☐ Type 1 ☐ Type 2 ☐ Type 3<br>☐ Type 4 ☐ Type 5 ☐Type 6 ☐ Type 7
Time of collected stool sample	☐☐:☐☐ ☐ 24 hour clock ☐ AM ☐ PM
Did this sample come into contact with diaper cream?	☐ yes, brand:___________________________ ☐ no
This sample was collected from a (select one):	☐ disposable diaper ☐ cloth diaper ☐ potty ☐ toilet ☐ other
Number of bowel movements during this day	☐☐ bowel movements
Other comments (e.g. infections, health complaints)	
If you are currently breastfeeding, please list any prescription medications that you took today.	
How much time did your baby spend in sleep last night between 7pm and 7am?	☐☐ hours and ☐☐ minutes
How many times did your baby wake up last night (7pm to 7am)?	☐☐ times
How much time did your baby spend in wakefulness between 10pm and 6am?	☐☐ hours and ☐☐ minutes
Please indicate if your baby had any interaction today with:	☐ Siblings ☐ Other children ☐ Animals that stay inside the house<br>☐ Animals that go outside and inside the house ☐Animals that stay outside the house

Day 10

	Quantity	Food or Drink	Processing	Brand	Tolerated?
**Feeding 1**<br><br>Time:					
**Feeding 2**<br><br>Time:					
**Feeding 3**<br><br>Time:					
**Feeding 4**<br><br>Time:					
**Feeding 5**<br><br>Time:					
**Feeding 6**<br><br>Time:					

Day 10

	Quantity	Food or Drink	Processing	Brand	Tolerated?
**Feeding 7**<br><br>Time:					
**Feeding 8**<br><br>Time:					
**Feeding 9**<br><br>Time:					

	Quantity	Name	Number of doses	Brand	Route (oral, IV, topical etc)
**Medication or vitamin 1**					
**Medication or vitamin 2**					
**Medication or vitamin 3**					
**Medication or vitamin 4**					
**Medication or vitamin 5**					

Day 11

Date	day ☐☐ month ☐☐ 20☐☐
Infant fecal sample collected	☐ yes ☐ no
Stool consistency of collected infant fecal sample (see Bristol Stool Chart)	☐ Type 1 ☐ Type 2 ☐ Type 3<br>☐ Type 4 ☐ Type 5 ☐Type 6 ☐ Type 7
Time of collected stool sample	☐☐:☐☐ ☐ 24 hour clock ☐ AM ☐ PM
Did this sample come into contact with diaper cream?	☐ yes, brand:___________________________ ☐ no
This sample was collected from a (select one):	☐ disposable diaper ☐ cloth diaper ☐ potty ☐ toilet ☐ other
Number of bowel movements during this day	☐☐ bowel movements
Other comments (e.g. infections, health complaints)	
If you are currently breastfeeding, please list any prescription medications that you took today.	

How much time did your baby spend in sleep last night between 7pm and 7am?	☐☐ hours and ☐☐ minutes
How many times did your baby wake up last night (7pm to 7am)?	☐☐ times
How much time did your baby spend in wakefulness between 10pm and 6am?	☐☐ hours and ☐☐ minutes

Please indicate if your baby had any interaction today with:	☐ Siblings ☐ Other children ☐ Animals that stay inside the house<br>☐ Animals that go outside and inside the house ☐Animals that stay outside the house

Day 11

	Quantity	Food or Drink	Processing	Brand	Tolerated?
**Feeding 1**<br>Time:					
**Feeding 2**<br>Time:					
**Feeding 3**<br>Time:					
**Feeding 4**<br>Time:					
**Feeding 5**<br>Time:					
**Feeding 6**<br>Time:					

Day 11

	Quantity	Food or Drink	Processing	Brand	Tolerated?
**Feeding 7**<br><br>Time:					
**Feeding 8**<br><br>Time:					
**Feeding 9**<br><br>Time:					

	Quantity	Name	Number of doses	Brand	Route (oral, IV, topical etc)
**Medication or vitamin 1**					
**Medication or vitamin 2**					
**Medication or vitamin 3**					
**Medication or vitamin 4**					
**Medication or vitamin 5**					

Day 12

Date	day ☐☐ month ☐☐ 20☐☐
Infant fecal sample collected	☐ yes ☐ no
Stool consistency of collected infant fecal sample (see Bristol Stool Chart)	☐ Type 1 ☐ Type 2 ☐ Type 3<br>☐ Type 4 ☐ Type 5 ☐Type 6 ☐ Type 7
Time of collected stool sample	☐☐:☐☐ ☐ 24 hour clock ☐ AM ☐ PM
Did this sample come into contact with diaper cream?	☐ yes, brand:___________________________ ☐ no
This sample was collected from a (select one):	☐ disposable diaper ☐ cloth diaper ☐ potty ☐ toilet ☐ other
Number of bowel movements during this day	☐☐ bowel movements
Other comments (e.g. infections, health complaints)	
If you are currently breastfeeding, please list any prescription medications that you took today.	

How much time did your baby spend in sleep last night between 7pm and 7am?	☐☐ hours and ☐☐ minutes
How many times did your baby wake up last night (7pm to 7am)?	☐☐ times
How much time did your baby spend in wakefulness between 10pm and 6am?	☐☐ hours and ☐☐ minutes

Please indicate if your baby had any interaction today with:	☐ Siblings ☐ Other children ☐ Animals that stay inside the house<br>☐ Animals that go outside and inside the house ☐Animals that stay outside the house

Day 12

	Quantity	Food or Drink	Processing	Brand	Tolerated?
**Feeding 1**<br><br>Time:					
**Feeding 2**<br><br>Time:					
**Feeding 3**<br><br>Time:					
**Feeding 4**<br><br>Time:					
**Feeding 5**<br><br>Time:					
**Feeding 6**<br><br>Time:					

Day 12

	Quantity	Food or Drink	Processing	Brand	Tolerated?
**Feeding 7**<br><br>Time:					
**Feeding 8**<br><br>Time:					
**Feeding 9**<br><br>Time:					

	Quantity	Name	Number of doses	Brand	Route (oral, IV, topical etc)
**Medication or vitamin 1**					
**Medication or vitamin 2**					
**Medication or vitamin 3**					
**Medication or vitamin 4**					
**Medication or vitamin 5**					

Day 13

Date	day ☐☐ month ☐☐ 20☐☐	
Infant fecal sample collected	☐ yes ☐ no	
Stool consistency of collected infant fecal sample (see Bristol Stool Chart)	☐ Type 1 ☐ Type 2 ☐ Type 3<br>☐ Type 4 ☐ Type 5 ☐Type 6 ☐ Type 7	
Time of collected stool sample	☐☐:☐☐ ☐ 24 hour clock ☐ AM ☐ PM	
Did this sample come into contact with diaper cream?	☐ yes, brand:____________________________ ☐ no	
This sample was collected from a (select one):	☐ disposable diaper ☐ cloth diaper ☐ potty ☐ toilet ☐ other	
Number of bowel movements during this day	☐☐ bowel movements	
Other comments (e.g. infections, health complaints)		
If you are currently breastfeeding, please list any prescription medications that you took today.		
How much time did your baby spend in sleep last night between 7pm and 7am?		☐☐ hours and ☐☐ minutes
How many times did your baby wake up last night (7pm to 7am)?		☐☐ times
How much time did your baby spend in wakefulness between 10pm and 6am?		☐☐ hours and ☐☐ minutes
Please indicate if your baby had any interaction today with:	☐ Siblings ☐ Other children ☐ Animals that stay inside the house<br>☐ Animals that go outside and inside the house ☐Animals that stay outside the house	

Day 13

	Quantity	Food or Drink	Processing	Brand	Tolerated?
**Feeding 1**<br><br>Time:					
**Feeding 2**<br><br>Time:					
**Feeding 3**<br><br>Time:					
**Feeding 4**<br><br>Time:					
**Feeding 5**<br><br>Time:					
**Feeding 6**<br><br>Time:					

Day 13

	Quantity	Food or Drink	Processing	Brand	Tolerated?
**Feeding 7**<br><br>Time:					
**Feeding 8**<br><br>Time:					
**Feeding 9**<br><br>Time:					

	Quantity	Name	Number of doses	Brand	Route (oral, IV, topical etc)
**Medication or vitamin 1**					
**Medication or vitamin 2**					
**Medication or vitamin 3**					
**Medication or vitamin 4**					
**Medication or vitamin 5**					

Day 14

Date	day ☐☐ month ☐☐ 20☐☐
Infant fecal sample collected	☐ yes ☐ no
Stool consistency of collected infant fecal sample (see Bristol Stool Chart)	☐ Type 1 ☐ Type 2 ☐ Type 3<br>☐ Type 4 ☐ Type 5 ☐Type 6 ☐ Type 7
Time of collected stool sample	☐☐:☐☐ ☐ 24 hour clock ☐ AM ☐ PM
Did this sample come into contact with diaper cream?	☐ yes, brand:_________________________ ☐ no
This sample was collected from a (select one):	☐ disposable diaper ☐ cloth diaper ☐ potty ☐ toilet ☐ other
Number of bowel movements during this day	☐☐ bowel movements
Other comments (e.g. infections, health complaints)	
If you are currently breastfeeding, please list any prescription medications that you took today.	

How much time did your baby spend in sleep last night between 7pm and 7am?	☐☐ hours and ☐☐ minutes
How many times did your baby wake up last night (7pm to 7am)?	☐☐ times
How much time did your baby spend in wakefulness between 10pm and 6am?	☐☐ hours and ☐☐ minutes

Please indicate if your baby had any interaction today with:	☐ Siblings ☐ Other children ☐ Animals that stay inside the house<br>☐ Animals that go outside and inside the house ☐Animals that stay outside the house

Day 14

	Quantity	Food or Drink	Processing	Brand	Tolerated?
**Feeding 1**<br><br>Time:					
**Feeding 2**<br><br>Time:					
**Feeding 3**<br><br>Time:					
**Feeding 4**<br><br>Time:					
**Feeding 5**<br><br>Time:					
**Feeding 6**<br><br>Time:					

Day 14

	Quantity	Food or Drink	Processing	Brand	Tolerated?
**Feeding 7**<br><br>Time:					
**Feeding 8**<br><br>Time:					
**Feeding 9**<br><br>Time:					

	Quantity	Name	Number of doses	Brand	Route (oral, IV, topical etc)
**Medication or vitamin 1**					
**Medication or vitamin 2**					
**Medication or vitamin 3**					
**Medication or vitamin 4**					
**Medication or vitamin 5**					

Day 15

Date	day ☐☐ month ☐☐ 20☐☐
Infant fecal sample collected	☐ yes ☐ no
Stool consistency of collected infant fecal sample (see Bristol Stool Chart)	☐ Type 1 ☐ Type 2 ☐ Type 3<br>☐ Type 4 ☐ Type 5 ☐Type 6 ☐ Type 7
Time of collected stool sample	☐☐:☐☐ ☐ 24 hour clock ☐ AM ☐ PM
Did this sample come into contact with diaper cream?	☐ yes, brand:___________________________ ☐ no
This sample was collected from a (select one):	☐ disposable diaper ☐ cloth diaper ☐ potty ☐ toilet ☐ other
Number of bowel movements during this day	☐☐ bowel movements
Other comments (e.g. infections, health complaints)	
If you are currently breastfeeding, please list any prescription medications that you took today.	

How much time did your baby spend in sleep last night between 7pm and 7am?	☐☐ hours and ☐☐ minutes
How many times did your baby wake up last night (7pm to 7am)?	☐☐ times
How much time did your baby spend in wakefulness between 10pm and 6am?	☐☐ hours and ☐☐ minutes

Please indicate if your baby had any interaction today with:	☐ Siblings ☐ Other children ☐ Animals that stay inside the house<br>☐ Animals that go outside and inside the house ☐Animals that stay outside the house

Day 15

	Quantity	Food or Drink	Processing	Brand	Tolerated?
**Feeding 1**					
Time:					
**Feeding 2**					
Time:					
**Feeding 3**					
Time:					
**Feeding 4**					
Time:					
**Feeding 5**					
Time:					
**Feeding 6**					
Time:					

Day 15

	Quantity	Food or Drink	Processing	Brand	Tolerated?
**Feeding 7**<br><br>Time:					
**Feeding 8**<br><br>Time:					
**Feeding 9**<br><br>Time:					

	Quantity	Name	Number of doses	Brand	Route (oral, IV, topical etc)
**Medication or vitamin 1**					
**Medication or vitamin 2**					
**Medication or vitamin 3**					
**Medication or vitamin 4**					
**Medication or vitamin 5**					

Day 16

Date	day ☐☐ month ☐☐ 20☐☐
Infant fecal sample collected	☐ yes ☐ no
Stool consistency of collected infant fecal sample (see Bristol Stool Chart)	☐ Type 1 ☐ Type 2 ☐ Type 3<br>☐ Type 4 ☐ Type 5 ☐Type 6 ☐ Type 7
Time of collected stool sample	☐☐:☐☐ ☐ 24 hour clock ☐ AM ☐ PM
Did this sample come into contact with diaper cream?	☐ yes, brand:___________________________ ☐ no
This sample was collected from a (select one):	☐ disposable diaper ☐ cloth diaper ☐ potty ☐ toilet ☐ other
Number of bowel movements during this day	☐☐ bowel movements
Other comments (e.g. infections, health complaints)	
If you are currently breastfeeding, please list any prescription medications that you took today.	
How much time did your baby spend in sleep last night between 7pm and 7am?	☐☐ hours and ☐☐ minutes
How many times did your baby wake up last night (7pm to 7am)?	☐☐ times
How much time did your baby spend in wakefulness between 10pm and 6am?	☐☐ hours and ☐☐ minutes
Please indicate if your baby had any interaction today with:	☐ Siblings ☐ Other children ☐ Animals that stay inside the house<br>☐ Animals that go outside and inside the house ☐Animals that stay outside the house

Day 16

	Quantity	Food or Drink	Processing	Brand	Tolerated?
**Feeding 1**<br><br>Time:					
**Feeding 2**<br><br>Time:					
**Feeding 3**<br><br>Time:					
**Feeding 4**<br><br>Time:					
**Feeding 5**<br><br>Time:					
**Feeding 6**<br><br>Time:					

Day 16

	Quantity	Food or Drink	Processing	Brand	Tolerated?
**Feeding 7**<br><br>Time:					
**Feeding 8**<br><br>Time:					
**Feeding 9**<br><br>Time:					

	Quantity	Name	Number of doses	Brand	Route (oral, IV, topical etc)
**Medication or vitamin 1**					
**Medication or vitamin 2**					
**Medication or vitamin 3**					
**Medication or vitamin 4**					
**Medication or vitamin 5**					

Day 17

Date	day ☐☐ month ☐☐ 20☐☐
Infant fecal sample collected	☐ yes ☐ no
Stool consistency of collected infant fecal sample (see Bristol Stool Chart)	☐ Type 1 ☐ Type 2 ☐ Type 3<br>☐ Type 4 ☐ Type 5 ☐Type 6 ☐ Type 7
Time of collected stool sample	☐☐:☐☐ ☐ 24 hour clock ☐ AM ☐ PM
Did this sample come into contact with diaper cream?	☐ yes, brand:___________________________ ☐ no
This sample was collected from a (select one):	☐ disposable diaper ☐ cloth diaper ☐ potty ☐ toilet ☐ other
Number of bowel movements during this day	☐☐ bowel movements
Other comments (e.g. infections, health complaints)	
If you are currently breastfeeding, please list any prescription medications that you took today.	

How much time did your baby spend in sleep last night between 7pm and 7am?	☐☐ hours and ☐☐ minutes
How many times did your baby wake up last night (7pm to 7am)?	☐☐ times
How much time did your baby spend in wakefulness between 10pm and 6am?	☐☐ hours and ☐☐ minutes

Please indicate if your baby had any interaction today with:	☐ Siblings ☐ Other children ☐ Animals that stay inside the house<br>☐ Animals that go outside and inside the house ☐Animals that stay outside the house

Day 17

	Quantity	Food or Drink	Processing	Brand	Tolerated?
**Feeding 1**<br>Time:					
**Feeding 2**<br>Time:					
**Feeding 3**<br>Time:					
**Feeding 4**<br>Time:					
**Feeding 5**<br>Time:					
**Feeding 6**<br>Time:					

Day 17

	Quantity	Food or Drink	Processing	Brand	Tolerated?
**Feeding 7**<br><br>Time:					
**Feeding 8**<br><br>Time:					
**Feeding 9**<br><br>Time:					

	Quantity	Name	Number of doses	Brand	Route (oral, IV, topical etc)
**Medication or vitamin 1**					
**Medication or vitamin 2**					
**Medication or vitamin 3**					
**Medication or vitamin 4**					
**Medication or vitamin 5**					

THE BRISTOL STOOL FORM SCALE (for children)

choose your POO!

type 1	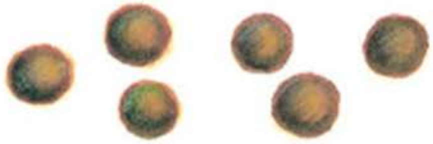	looks like: **rabbit droppings** Separate hard lumps, like nuts (hard to pass)
type 2	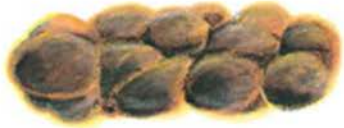	looks like: **bunch of grapes** Sausage-shaped but lumpy
type 3	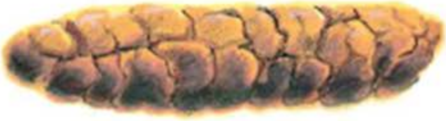	looks like: **corn on cob** Like a sausage but with cracks on its surface
type 4	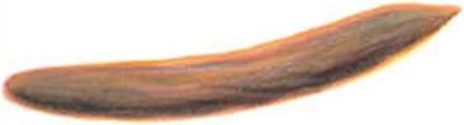	looks like: **sausage** Like a sausage or snake, smooth and soft
type 5	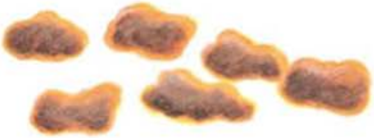	looks like: **chicken nuggets** Soft blobs with clear-cut edges (passed easily)
type 6	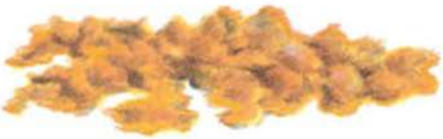	looks like: **porridge** Fluffy pieces with ragged edges, a mushy stool
type 7	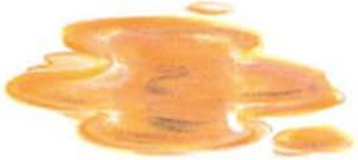	looks like: **gravy** Watery, no solid pieces ENTIRELY LIQUID
