# Supplementary material for: Investigating colonization patterns of the infant gut microbiome during the introduction of solid food and weaning from breastmilk: A cohort study protocol
Source: PLoS One. 2021 Apr 2;16(4):e0248924. doi: 10.1371/journal.pone.0248924 (PMC8018627; doi:10.1371/journal.pone.0248924)
Supplement: Attachment — Submitted filename: Response to Reviewers.docx [file pone.0248924.s003.docx]

Editor Comments:

As pointed out by the reviewer, this protocol reports lacks a few important points:

Is the sample size justified by power calculations from previous studies? There are enough studies that have followed longitudinally babies gut microbiome to perform such power calculations, or at least support what seems to be a relatively small number of participants.

Thank you for pointing out this oversight. We have added the following statement to the methods section of our manuscript:

“This study focuses on within-subject differences in microbiota composition whereby each individual serves as its own control, an approach that increases power relative to studies comparing differences among individuals. With this study design, for an estimated expected mean of paired differences of 10 observed species and an expected standard deviation of the paired differences of 8, a sample size of 9 infants in each cohort is required to achieve a power of 80% and a level of significance of 5% (two sided).”

The manuscript would benefit from a "hypotheses and potential outcomes" section. It is difficult to understand why the study design, apart that it is (supposedly) different from what has been done before.

We have added the following in our Introduction:

“We hypothesize that during the introduction to solid food and during the time around weaning from breastmilk we will see a shift in microbial composition to be more complex and less dominated by Bifidobacterium., We expect to see changes in the stool microbiome and metabolome within 14 days of each dietary change. Hhowever since little is known about how these dietary changes will impact the ecology of the gut microbiome, this work is largely hypothesis-generating rather than hypothesis-testing.”

We have also elaborated on our outcomes of interest in the Outcome Assessment section:

“ We expect that bacterial richness and diversity will increase over time and as the diet becomes more complex in both the full-term and the preterm infants. In the full-term cohort we expect there to be a loss of breastfeeding associated taxa such as some species of Bifidobacterium, that have been observed to decrease over the first year of life and to differ with breastfeeding status (Pannaraj et al., 2017 ; Henrich et, al., 2018 ). We expect to describe significant shifts in the overall microbial community membership and substrate use over the study periods through prediction of bacterial genes and the abundance of metabolites in the stool that differ based on geography, birth mode and diet.”

The authors state that daily sampling will be superior to detect fine changes. However, a case could be made that especially for babies/infants, significant stochasticity in microbiome dynamics is possible. This should at least be discussed.

We recognize that that daily sampling may reveal stochasticity and that our methods may not necessarily be superior to wider sampling intervals. Instead, we think that both strategies are valuable and contribute different information to this field of research. We have modified what we had originally written in the Discussion to reflect this. In addition, we have provided a more in depth explanation for rationale for daily sampling in the Discussion and here:

In order to capture the potential change in microbiome as it occurs, we are asking participants to collect stool samples daily (where possible). A [2020 review](https://www.frontiersin.org/articles/10.3389/fnut.2020.00079/full) of study design methods to evaluate the diet and microbiome relationship suggested that dense, longitudinal sampling with samples collected before, during and after an intervention of interest is ideal. We are attempting to accomplish both of these by asking participants to collect daily fecal samples over a 14 to 17 day period that spans from before to after the introduction of solid foods and again at weaning. We realize that it is quite possible that many of our participants will not be able to achieve daily sampling for the entire sampling period. The review states that up to 7 to 9 sequential samples is ideal in order to account for intra-individual variation and we feel that by striving to obtain samples over a 14 to 17 day period we will meet this target.

Reviewers' comments:

1. Does the manuscript provide a valid rationale for the proposed study, with clearly identified and justified research questions?

The research question outlined is expected to address a valid academic problem or topic and contribute to the base of knowledge in the field.

Reviewer #1: Yes

2. Is the protocol technically sound and planned in a manner that will lead to a meaningful outcome and allow testing the stated hypotheses?

The manuscript should describe the methods in sufficient detail to prevent undisclosed flexibility in the experimental procedure or analysis pipeline, including sufficient outcome-neutral conditions (e.g. necessary controls, absence of floor or ceiling effects) to test the proposed hypotheses and a statistical power analysis where applicable. As there may be aspects of the methodology and analysis which can only be refined once the work is undertaken, authors should outline potential assumptions and explicitly describe what aspects of the proposed analyses, if any, are exploratory.

Reviewer #1: Partly

3. Is the methodology feasible and described in sufficient detail to allow the work to be replicable?

Reviewer #1: Yes

4. Have the authors described where all data underlying the findings will be made available when the study is complete?

The [PLOS Data policy](https://journals.plos.org/plosone/s/materials-and-software-sharing) requires authors to make all data underlying the findings described in their manuscript fully available without restriction, with rare exception, at the time of publication. The data should be provided as part of the manuscript or its supporting information, or deposited to a public repository. For example, in addition to summary statistics, the data points behind means, medians and variance measures should be available. If there are restrictions on publicly sharing data—e.g. participant privacy or use of data from a third party—those must be specified.

Reviewer #1: No

5. Is the manuscript presented in an intelligible fashion and written in standard English?

PLOS ONE does not copyedit accepted manuscripts, so the language in submitted articles must be clear, correct, and unambiguous. Any typographical or grammatical errors should be corrected at revision, so please note any specific errors here.

Reviewer #1: Yes

6. Review Comments to the Author

Please use the space provided to explain your answers to the questions above and, if applicable, provide comments about issues authors must address before this protocol can be accepted for publication. You may also include additional comments for the author, including concerns about research or publication ethics.

You may also provide optional suggestions and comments to authors that they might find helpful in planning their study.


(Please upload your review as an attachment if it exceeds 20,000 characters)

Reviewer #1: Overall:
No real hypothesis specified
Outcomes measures vague
Exact relationships between cessation of breastfeeding in full-term and pre-term infants and gut microbiome are not specified, therefore the study seems to be more of a simple observation than a hypothesis-testing scientific study.


Several grammatical errors and problems:

Thank you for pointing out these details. We have made all of the suggested changes below.

Lines 39-41 confusing, suggest re-writing
May need to hyphenate “full-term,” “adult-like,” and “evidence-based”
Line 52-need to indicate in the sentence that the study will detail effects of transition ON MICROBIOME
Line 66 need the word “and” after the word “species”
Line 79 need a comma before the word “and”
Line 81 need a comma after the word “infants”
Line 93, hyphenate “diet-related”
Consistently use “pre-term” or “preterm” or “pre term,” same goes for full term
Line 97-hyphenate “animal-based”
Line 100 hyphenate “culture-based”
Line 112, need the word “and” after “vitro”

Line 114, need comma after the word “during”
Line 124, remove the hyphen in “take part”
Sentence that spans line 140-142 needs to be edited for grammar, it does not make sense
Table 1 is confusing , should probably be edited down.
Table 1 row 2 should be the same in both the primary and IS cohorts columns
In table 1, there is a typo. Written is “Total Intensively Sample sub-study sample size…” it should say “Total Intensively Sampled…” this happens a few times

Line 262- need to hyphenate “14-day” and also make clear that there are 14-day and 17-day sampling periods (or change protocol so that sampling periods are the same in all subsamples)

Other issues:

Multiple births at all are excluded from the full-term sub-sample cohorts, but it seems as though twins are allowed in the Baby&Pre-Mi cohort. Is this the case? If so, it shouldn’t be that way, and if not, the paragraph starting on line 167 needs to be edited to clarify.

Multiple gestations were excluded from the full term cohorts. The preterm cohort did include multiple gestations (twins). These were the eligibility criteria of the source cohorts (LucKi, Baby & Mi and Baby & Pre-Mi) which were chosen for clinical reasons relevant to the primary objectives of the source cohorts. We could have excluded multiple gestations from this intensively sampled subset of preterm infants but chose not to in order to maximize the number of participants that would be eligible for this sub-study. We recognize that the inclusion of twin participants reduces their statistical independence however, the collection of samples before, during and after each dietary change allows each infant to serve as their own baseline comparison. This follows the methodology of previous high impact studies of preterm infants which included multiple gestations (Xiong et al, 2017 and Raveh-Sadka et al, 2016).

Xiong W, Brown CT, Morowitz MJ, Banfield JF, Hettich RL. Genome-resolved metaproteomic characterization of preterm infant gut microbiota development reveals species-specific metabolic shifts and variabilities during early life. Microbiome. 2017 Dec;5(1):1-3.

Raveh-Sadka T, Firek B, Sharon I, Baker R, Brown CT, Thomas BC, Morowitz MJ, Banfield JF. Evidence for persistent and shared bacterial strains against a background of largely unique gut colonization in hospitalized premature infants. The ISME journal. 2016 Dec;10(12):2817-30.

Line 183 – why won’t pre-term infants be included in the weaning sampling frame? They should be.

Preterm infants will not be included in the weaning sampling time frame because breastfeeding rates in the preterm population are lower than among full terms. By sampling from only those that breastfed we would be creating a biased cohort and our findings would not be generalizable to the general preterm population. We have clarified this in the manuscript.

Line 188-192 -why are there different lengths of sample collection between baby+mi and baby+premi and lucki participants? Shouldn’t they be the same length?

This study is a collaboration between three existing cohorts. As a result of varying resources and situations, the duration of sample collection between cohorts differs. We do not feel that this will impact our ability to compare results between cohorts.

Outcome assessment – need to describe whether they believe outcomes will increase or decrease over time and based on what factors, and whether there will be differences between their subsamples and why.

See reply above to the request for a “Hypothesis and Outcome” section.

7. PLOS authors have the option to publish the peer review history of their article ([what does this mean?](https://journals.plos.org/plosone/s/editorial-and-peer-review-process#loc-peer-review-history)). If published, this will include your full peer review and any attached files.


If you choose “no”, your identity will remain anonymous but your review may still be made public.


**Do you want your identity to be public for this peer review?** For information about this choice, including consent withdrawal, please see our [Privacy Policy](https://www.plos.org/privacy-policy).

Reviewer #1: No
